# 25-hydroxyvitamin D (25-OHD) levels in Turkish geriatric population: A nationwide study

**DOI:** 10.5937/jomb0-36921

**Published:** 2022-10-15

**Authors:** Murat Çağlayan, Cigdem Sonmez, Mehmet Senes, Ataman Gonel, Ozlem Gulbahar, Nurbanu Bursa, Derun Taner, Osman Celik, Fidanci Ali Aykut, Ulgu Mustafa Mahir, Abdulvahit Sozuer, Naim Ata, Suayip Birinci

**Affiliations:** 1 Health Science University, Diskapi Yildirim Beyazit Training and Research Hospital, Department of Medical Biochemistry, Ankara, Turkey; 2 Dr. Abdurrahman Yurtaslan Ankara Oncology Training and Research Hospital, Department of Medical Biochemistry, Ankara, Turkey; 3 Ankara Training and Research Hospital, Department of Medical Biochemistry, Ankara, Turkey; 4 HasanKalyoncu University, Faculty of Health Science, Department of Nutrition and Dietetics, Gaziantep, Turkey; 5 Gazi University Training And Research Hospital, Department of Medical Biochemistry, Ankara, Turkey; 6 Hacettepe University, Department of Statistics, Ankara, Turkey; 7 Keciören Training and Research Hospital, Department of Internal Medicine, Ankara, Turkey; 8 Republic of Turkey Ministry of Health, Ankara, Turkey; 9 Istanbul Provincial Health Department, Istanbul, Turkey

**Keywords:** 25-OH vitamin D, geriatry, big data, seasons, vitamin D deficiency, sunbathing time, 25-OH vitamin D, gerijatrija, veliki podaci, godišnja doba, nedostatak vitamina D, vreme sunčanja

## Abstract

**Background:**

Across the world, 25-hydroxyvitamin D (25-OHD) deficiency is a major health problem associated with many chronic diseases in the geriatric population. Prior to this study, there were no data regarding 25-OHD levels among individuals over the age of 65 in Turkey. The aim of this study was to assess 25-OHD levels and seasonal variations in these values among people over the age of 65 in Turkey.

**Methods:**

This study included vitamin D measurements taken in 2016, 2017, and 2018 from the Turkish population over the age of 65. The age, gender, and seasonal average data of the study population were defined. The study data were obtained from the database of the Ministry of Health, and a Kolmogorov-Smirnov test was used to assess the distribution of the data. Medians and interquartile ranges (IQRs) were calculated for all categories, as the data were nonparametric.

**Results:**

The number of vitamin D measurements taken from the geriatric individuals included in this study was 305,329 for 2016, 576,452 for 2017, and 752,837 for 2018. The medians and IQRs of the 25-OHD levels in this population were 16 μg/L (IQR 7.45-24.55 μg/L) for 2016, 16.1 μg/L (IQR 7.8-24.4 μg/L) for 2017, and 16.4 μg/L (IQR 8.95-23.85 μg/L) for 2018.

**Conclusions:**

While the 25-OHD levels of older men tended to increase during the period of seasonal sunlight in Turkey, this variability was observed in elderly women. This suggests that older women tend to live more sedentary lives and have insufficient sun exposure. Overall, the median 25-OHD levels of individuals over the age of 65 tended to decrease each year.

## Abstract (sr)

**Uvod:**

Širom sveta, nedostatak 25-hidroksivitamina D (25-OHD) je veliki zdravstveni problem povezan sa mnogim hroničnim bolestima u gerijatrijskoj populaciji. Pre ove studije, nije bilo podataka o nivoima 25-OHD me u pojedincima starijim od 65 godina u Turskoj. Cilj ove studije je bio da se proceni nivo 25-OHD i sezonske varijacije ovih vrednosti kod ljudi starijih od 65 godina u Turskoj.

**Metode:**

Ova studija je uključila merenja vitamina D uzeta 2016, 2017. i 2018. godine od turske populacije starije od 65 godina. Definisani su podaci o starosti, polu i sezonskom proseku ispitivane populacije. Podaci studije su dobijeni iz baze podataka Ministarstva zdravlja, a za procenu distribucije podataka korišćen je Kolmogorov-Smirnov test. Medijani i interkvartilni rasponi (IKR) su izračunati za sve kategorije, pošto su podaci bili neparametrijski.

**Rezultati:**

Broj merenja vitamina D uzetih od gerijatrijskih pojedinaca uključenih u ovu studiju bio je 305.329 za 2016, 576.452 za 2017. i 752.837 za 2018. Medijani i IKR nivoa 25-OHD u ovoj populaciji su bili 16 mg/L IKR 7,45-24,55 mg/L) za 2016, 16,1 mg/L (IKR 7,8-24,4 mg/L) za 2017, i 16,4 mg/L (IKR 8,95-23,85 mg/L) za 2018.

**Zaključak:**

Dok je nivo 25-OHD kod starijih muškaraca imao tendenciju povećanja tokom perioda sezonske sunčeve svetlosti u Turskoj, ova varijabilnost je primećena kod starijih žena. Ovo sugeriše da starije žene imaju tendenciju da žive više sedentarno i da su nedovoljno izložene suncu. Sve u svemu, srednji nivoi 25-OHD kod osoba starijih od 65 godina su se smanjivale svake godine.

## Introduction

Vitamin D deficiency, while common in all age groups, is most frequently observed in older individuals [1]. Risk factors for vitamin D deficiency include insufficient exposure to sunlight, inadequate dietary intake and supplementation, obesity, drug use, sunscreen use, skin pigmentation, and advanced age [2]. In the geriatric population, immobility reduces exposure to sunlight and the capacity for 25-hydroxyvitamin D (25-OHD) production in the skin [3]. Even with the same amount of sunlight exposure, a 70-year-old person produces approximately 25% of the vitamin D3 produced by a 20-year-old counterpart [4].

Prior studies have revealed that many problems occur in older adults due to 25-OHD deficiency, such as an increased risk of falling, impaired lower extremity function, lower muscle strength, and reduced bone mineralization [5] [6] [7]. Insufficient or deficient 25-OHD is associated with many chronic conditions, including cardiovascular diseases, obesity, metabolic syndrome, diabetes mellitus, infections, autoimmune diseases, and some malignancies [8] [9] [10] [11]. Therefore, the detection and treatment of vitamin D deficiency in older people can be indirectly important for the prevention of these chronic conditions.

The aim of this study was to evaluate the 25-OHD levels and seasonal 25-OHD variations of Turkish individuals aged ≥65 years.

## Materıals and methods

The health records of individuals aged ≥65 years who were admitted to public, private, and university health institutions were collected via the e-health database of the Turkish Ministry of Health. This e-health database includes two headings: laboratory service information and test process information. The test process information consists of the test name, test result, test unit, and reference range, whereas the laboratory service information includes the demographics of the individuals. This study included 25-OHD measurements from 2016-2018. Ages were grouped as 65-74, 75-84, and 85 years. This study was conducted according to the Declaration of Helsinki and received approval from the Turkish Ministry of Health with a waiver of informed consent for retrospective data analysis (95741342-020/27112019).

In Turkey, 25-OHD is analyzed using an immunoassay method or other chromatographic techniques. All units of measurement are used as μg/L. In particular, most Turkish institutes (approximately 95%) use immunochemical methods. The number of requested 25-OHD measurements was 305,219 for 2016, 576,452 for 2017, and 752,837 for 2018. For this study, 25-OHD levels ≥150 μg/L were excluded. The statistical analysis was conducted using SPSS (version 20), and a Kolmogorov-Smirnov test was used to assess the distribution of the data. Since the distribution of the data was nonparametric, medians and interquartile ranges (IQRs) were calculated for all categories. More specifically, the median 25-OHD level and IQR were calculated for each age group, and seasonal and monthly variations were evaluated for the gender groups and the total sample. The sunbathing time interval was taken from the official website of the Turkish State Meteorological Service [12].

## Results

The collected data showed the degree of 25-OHD deficiency and insufficiency in the elderly Turkish population. The median 25-OHD levels and IQRs of this population were 16 μg/L (IQR 7.45-24.55 μg/L) in 2016, 16.1 μg/L (IQR 7.8-24.4 μg/L) in 2017, and 16.4 μg/L (IQR 8.95-23.85 μg/L) in 2018. The medians, IQRs, and numbers are shown for all gender and age groups in Table 1. Inboth the gender groups and the total sample, 25-OHD levels decreased as age increased.

**Table 1:** The number of 25-OHD tests by the age and gender groups for the years 2016, 2017, 2018 (μg/L).

Year		Age Group	n	Median	IQR
2016	Male	65-74	52854	17.7	10.9-24.5
		75-84	28039	15.6	8.5-22.7
		85+	5741	12.8	5.7-19.9
		**Total **	**86634 **	**16.8 **	**9.75-23.85 **
	Female	65-74	139185	15.8	6.9-24.7
		75-84	64094	15.5	5.9-25.1
		85+	15306	13.1	3.6-22.6
		**Total **	**218585 **	**15.5 **	**6.35-24.65 **
	Total	65-74	192039	16.5	8.15-24.85
		75-84	92133	15.6	6.75-24.45
		85+	21047	13.0	4.2-21.8
		**Total **	**305219 **	**16.0 **	**7.45-24.55 **
2017	Male	65-74	106768	17.9	11.05-24.75
		75-84	56649	15.6	8.45-22.75
		85+	12313	12.9	5.6-20.2
		**Total **	**175730 **	**16.9 **	**8.25-27.1 **
	Female	65-74	252175	16.0	7.4-24.6
		75-84	117742	15.7	6.35-25.5
		85+	30804	13.4	4-22.8
		**Total **	**400721 **	**15.7 **	**6.85-24.55 **
	Total	65-74	358944	16.7	8.65-24.75
		75-84	174391	15.6	7.05-24.15
		85+	43117	13.2	3.25-22.6
		**Total **	**576452 **	**16.1 **	**7.8-24.4 **
2018	Male	65-74	145052	17.6	11.45-23.75
		75-84	71775	15.8	9.3-22.3
		85+	19296	13.2	6.45-19.95
		**Total **	**236123 **	**16.8 **	**10.4-23.2 **
	Female	65-74	325123	16.2	8.65-23.75
		75-84	148210	16.2	7.8-24.6
		85+	43379	14.2	5.55-22.85
		**Total **	**516712 **	**16.1 **	**7.45-24.25 **
	Total	65-74	470176	16.8	8.45-25.25
		75-84	219986	16.0	8.25-23.75
		85+	62675	13.9	5.85-21.95
		**Total **	**752837 **	**16.4 **	**8.95-23.85 **

**Figure 1:**
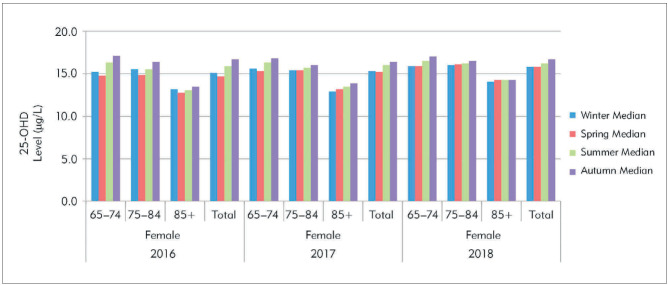
The histogram graphs of the 25-OHD by seasons in the female based on age groups between 2016–2018.

**Figure 2:**
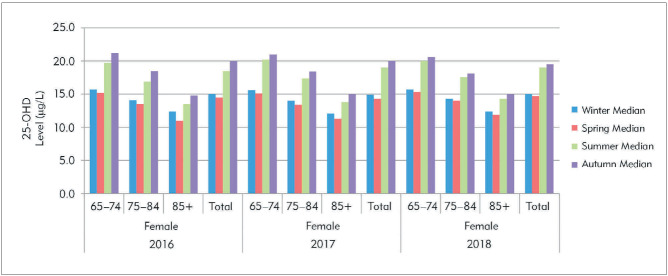
The histogram graphs of the 25-OHD by seasons in the male based on age groups between 2016–2018.

Seasonal variations in 25-OHD are shown for the gender groups and the total sample in Table 2. The 25-OHD levels of the male groups were more variable than those of the female groups. For both genders, 25-OHD levels were lower in winter and spring than in summer and autumn, as expected. The seasonal variations of the female and male groups are shown in the histograms of Figure 1 and Figure 2. Overall, seasonal variation was more apparent in the male groups. Rising 25-OHD levels in the summer and autumn were especially apparent in the group aged 65-74 years, whereas this increase was less obvious in the male group aged ≥85 years and the female groups at all ages.

**Table 2:** 25-OHD test numbers and results by seasons for the years 2016, 2017, 2018 (μg/L).

			Winter			Spring			Summer			Autumn		
			Count	Median	IQR	Count	Median	IQR	Count	Median	IQR	Count	Median	IQR
2016	Male	65–74	13570	15.7	9.3–22.1	15387	15.2	9.2–21.2	11417	19.7	13.25–26.15	12480	21.2	14.4–28
		75–84	6743	14.1	7.4–20.8	8209	13.5	7.05–19.95	6406	16.9	9.85–23.95	6681	18.5	11.3–25.7
		85+	1381	12.4	5.2–19.6	1640	11.0	4.65–17.35	1359	13.5	6.35–20.65	1361	14.8	7.65–21.95
		**Total**	**21694**	**15.0**	**8.4–21.6**	**25236**	**14.5**	**8.25–20.75**	**19182**	**18.5**	**11.65–25.35**	**20522**	**20.0**	**12.9–27.1**
	Female	65–74	35121	15.2	6.4–24	44229	14.8	5.5–24.1	28549	16.3	7.75–24.85	31286	17.1	8.5–25.7
		75–84	15506	15.5	5.85–25.15	19942	14.9	5.05–24.75	14181	15.5	6.05–24.95	14465	16.4	7–25.8
		85+	3667	13.2	3.5–22.9	4702	12.8	3–22.6	3606	13.1	3.95–22.25	3331	13.5	4.3–22.7
		**Total**	**54294**	**15.1**	**5.95–24.25**	**68873**	**14.7**	**5.2–24.2**	**46336**	**15.9**	**6.95–24.85**	**49082**	**16.7**	**7.8–25.6**
	Total	65–74	48691	15.4	7.35–23.45	59616	15.0	6.6–23.4	39966	17.5	9.4–25.6	43766	18.6	10.35–26.85
		75–84	22249	14.9	6.15–23.65	28151	14.3	5.4–23.2	20587	16.1	7.4–24.8	21146	17.2	8.45–25.9
		85+	5048	12.9	3.85–21.95	6342	12.0	3.1–20.9	4965	13.2	4.7–21.7	4692	14.1	5.55–22.65
		**Total**	**75988**	**15.1**	**6.75–23.45**	**94109**	**14.6**	**5.95–23.25**	**65518**	**16.8**	**8.4–25.2**	**69604**	**18.0**	**9.5–26.5**
2017	Male	65–74	27727	15.6	9.35–21.85	30706	15.1	8.8–21.4	22504	20.2	13.45–26.95	25831	21.0	14.5–27.5
		75–84	14163	14.0	7.3–20.7	16361	13.4	6.7–20.1	12853	17.4	10.15–24.65	13272	18.4	11.35–25.45
		85+	3006	12.1	5–19.2	3588	11.3	4.2–18.4	2862	13.8	6.5–21.1	2857	15.0	7.6–22.4
		**Total**	**44896**	**14.9**	**8.4–21.4**	**50655**	**14.3**	**7.75–20.85**	**38219**	**19.0**	**11.9–26.1**	**41960**	**20.0**	**13.15–26.85**
	Female	65–74	65074	15.6	6.85–24.35	77833	15.3	6.2–24.4	52432	16.3	7.95–24.65	56836	16.8	8.95–24.65
		75–84	29276	15.4	5.85–24.95	35883	15.4	5.45–25.35	25918	15.7	6.6–24.8	26665	16.0	7.55–24.45
		85+	7708	13.0	3.5–22.5	8957	13.2	3.2–23.2	7242	13.5	4.3–22.7	6897	13.9	5.1–22.7
		**Total**	**102058**	**15.3**	**6.25–24.35**	**12267**	**15.2**	**5.75–24.65**	**85592**	**16.0**	**7.3–24.7**	**90398**	**16.4**	**8.3–24.5**
	Total	65–74	92801	15.6	7.65–23.55	10853	15.2	6.95–23.45	74936	17.9	9.9–25.9	82668	18.4	10.75–26.05
		75–84	43439	14.8	6.25–23.35	52244	14.6	5.65–23.55	38771	16.4	7.9–24.9	39937	17.0	8.95–25.05
		85+	10714	12.6	3.65–21.55	12545	12.4	3.25–21.55	10104	13.7	5–22.4	9754	14.2	5.8–22.6
		**Total**	**146954**	**15.1**	**6.85–23.35**	**17332**	**14.9**	**6.35–23.45**	**12381**	**17.1**	**8.8–25.4**	**13235**	**17.7**	**9.85–25.55**
2018	Male	65–74	40947	15.7	9.8–21.6	41230	15.3	9.8–20.8	30076	20.0	14.25–25.75	32799	20.6	14.65–26.55
		75–84	19772	14.3	7.95–20.65	20582	14.0	7.9–20.1	15531	17.6	11.2–24	15890	18.1	11.55–24.65
		85+	5205	12.4	5.45–19.35	5553	11.9	5.6–18.2	4380	14.3	7.75–20.85	4158	15.0	8.35–21.65
		**Total**	**65924**	**15.0**	**8.85–21.15**	**67365**	**14.7**	**8.9–20.5**	**49987**	**19.0**	**12.85–25.15**	**52847**	**19.5**	**13.2–25.8**
	Female	65–74	93126	15.9	7.85–23.95	96132	15.9	8.05–23.75	65992	16.5	9.4–23.6	69873	17.0	9.85–24.1
		75–84	41085	16.0	7.2–24.8	43511	16.1	7.5–24.7	32274	16.2	8.2–24.2	31340	16.5	8.5–24.5
		85+	11844	14.1	5.05–23.15	12486	14.3	5.35–23.25	9922	14.3	6.05–22.55	9127	14.3	6.15–22.45
		**Total**	**146055**	**15.8**	**7.45–24.15**	**15212**	**15.8**	**7.65–23.95**	**10818**	**16.2**	**8.75–23.65**	**11034**	**16.7**	**9.15–24.25**
	Total	65–74	134074	15.8	8.45–23.15	13736	15.6	8.55–22.65	96068	17.9	11.15–24.65	10267	18.3	11.35–25.25
		75–84	60857	15.3	7.25–23.35	64093	15.2	9.2–21.2	47805	16.8	9.3–24.3	47231	17.1	9.55–24.65
		85+	17049	13.4	5–21.8	18039	13.4	7.05–19.95	14302	14.3	6.55–22.05	13285	14.5	6.8–22.2
		**Total**	**211980**	**15.5**	**7.85–23.15**	**21949**	**15.3**	**4.65–17.35**	**15817**	**17.3**	**10.2–24.4**	**16318**	**17.8**	**10.6–25**

**Figure 3:**
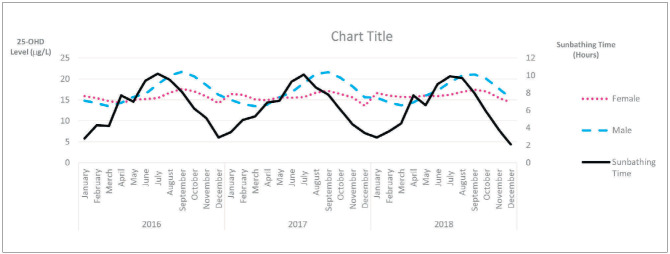
25-OHD levels and sunbathing time graphs according to gender and month between 2016–2018.

Seasonal variations in 25-OHD were evaluated in relation to sunbathing time by month. As indicated by the observed seasonal variations, sunbathing appeared to increase vitamin D synthesis more efficiently in the male groups than in the female groups. Increased 25-OHD levels were observed later than the increase in sunbathing time intervals. The sunbathing time intervals and 25-OHD levels of the male and female groups are shown in Figure 3.

## Dıscussıon

Vitamin D deficiency is common even along the Equator, which is the most sunlight-exposed region of the world [13] [14] [15]. More than a third of adults have been reported to have low 25-OHD levels, which are especially common in the geriatric population [16] [17]. This is the first comprehensive study to indicate serum 25-OHD levels in the elderly population (≥65 years old) of all regions of Turkey. In this study, the 25-OHD levels of this population in 2016, 2017, and 2018 were analyzed retrospectively and found to be deficient and insufficient. The study data were classified by year to evaluate the functionality of preventive measures for vitamin D deficiency, which is common in the geriatric population. From 2016 to 2018, it was observed that vitamin D levels increased in women, men, and the general population (Table 1). The median 25-OHD levels were 16 μg/L (IQR 7.45-24.55 μg/L) in 2016, 16.1 μg/L (IQR 7.8-24.4 μg/L) in 2017, and 16.4 μg/L (IQR 8.95-23.85 μg/L) in 2018 (Table 1).

Due to the unique properties of vitamin D synthesis, different serum levels have been monitored in distinct regions around the world, depending on geographical region, exposure to sunlight, skin color, and race. In recent years, interest in 25-OHD testing has increased worldwide. In some countries and even entire continents, 25-OHD levels are evaluated on the basis of physiological or disease state as well as demographic characteristics, such as gender, race, and age. Vitamin D deficiency has been observed across the world [9] [11]. Members of populations with low socioeconomic status especially breastfeeding babies, children, pregnant and premenopausal women, and older adults are considered to be at a high risk of vitamin D deficiency. In the present study, the 25-OHD levels detected in the older population of Turkey were lower than those found in other Middle Eastern countries for all gender and age groups [9] [11]. In Europe, vitamin D status varies according to latitude, season, and skin pigmentation. For example, 25-OHD levels are higher in Northern Europe than in Southern Europe and higher in Western Europe than in Eastern Europe. The high serum 25-OHD levels found in Norway and Sweden are most likely due to high fat intake from fish and cod liver oil. Meanwhile, the low vitamin D levels found in Spain, Italy, and Greece may be due to greater skin pigmentation and sun protection behavior [9].

One of the most comprehensive studies of vitamin D status in North America is the National Health and Nutrition Examination Survey (NHANES). According to the NHANES data, the average 25-OHD level of 4,495 individuals between 2005 and 2006 was 19.92 μg/L (20.12 μg/L in males and 19.8 μg/L in females). Furthermore, 25-OHD levels in North America decreased from 30 μg/L in 1988-1994 (n = 18,883) to 24 μg/L in 2001-2004 (n = 13,369) [9]. Average 25-OHD levels differ by country and are insufficient in most countries. In a 2015 study conducted in Hong Kong, China, 25-OHD levels were found to be 16.8-22.8 μg/L, 18.8-25.6 μg/L, and 16.4-22.4 μg/L in individuals aged 18-44, 45-64, and 65 years, respectively [18]. In a 2016 study of the USA, contrary to the present findings, 25-OHD levels were found to be 25.8 μg/L in the 20-39 age group (n = 3,349), 27.2 μg/L in the 40-59 age group (n = 3,377), and 28.4 μg/L in the 60 age group (n = 3,602) [19]. In another study conducted in New Zealand, 25-OHD levels were found to be 18.0 μg/L in the 18-50 age group (n = 154), 20.8 μg/L in the 50-64 age group (n = 130), and 20.5 μg/L in the 65-85 age group (n = 119) [20]. In the present study, the median 25-OHD levels of the female groups were lower than those of the male groups in all age groups except for the male group aged ≥85 years. In Jordan, a 2012 study of 2,032 females aged 15-50 years revealed an average 25-OHD level of 11 μg/L [21]. A 2010 study in the USA determined an average 25-OHD level of 23.6 μg/L among 5,173 females aged 13-44 [22]. With regard to 25-OHD levels in males, a study of 1,606 males aged 68-79 years in the USA showed that the average concentration of 25-OHD was 25.1 μg/L, higher than in the present study [23]. In a study conducted in Switzerland, the average 25-OHD concentration of 1,194 geriatric males was found to be 27.5 μg/L [24].

The present findings regarding seasonal variations in 25-OHD levels were as expected. The seasonal changes observed in the female groups were less than those observed in the male groups, except in those aged ≥85 years. In both the male and female 85 age groups, 25-OHD level variations were less than in the other age groups due to reduced mobilization and sunlight exposure. Changes in serum 25-OHD levels due to seasonal changes have been shown in some prior studies. In a prospective study of 42 female and 40 male healthy volunteers conducted by Costanzo et al. [25], seasonal variation was observed in serum 25-OHD levels, including a significant increase in both genders during the summer months. In both sexes, a positive correlation between exposure to sunlight and 25-OHD levels was found only in the winter season. In the present study, this correlation was detected in all seasons, especially in men. In a study of 576 women and 120 men conducted by Heidari et al. [26], the serum 25-OHD concentrations of women were found to be significantly lower than those of men in the summer and autumn, and similar results were found for all years in the present study. The observation of 25-OHD deficiency in areas with long durations of sunlight exposure suggests those reviewing measurement methods and some interferants, which may cause this situation [27] [28]. However, when the sunlight exposure of the geriatric population decreases, their vitamin D levels also stand to decrease, reducing these suspicions.

While the 25-OHD level distribution of men was similar to the curve of sunbathing duration, this similarity was not observed in women. This suggests that older women lack sufficient sunlight and lead more home-dependent, sedentary lifestyles. While the 25-OHD levels of men tended to increase in July, when sunbathing duration was at its highest level, the peak 25-OHD level was in September every year. Men tended to sunbathe less during hot months than in August and September, possibly to protect themselves from the harmful effects of hot weather. The 25-OHD levels of women did not correlate with sunbathing duration (Figure 3). The median 25-OHD levels of geriatric men and women and the total geriatric population tended to decline with each passing year. Furthermore, the average 25-OHD levels gradually decreased in the 65-74, 75-84, and 85 age groups (Figure 3). With regard to the seasons, median 25-OHD levels were found to be lower in the winter and spring across all age and gender groups (Table 2). The median 25-OHD levels of male individuals over the age of 65 peaked in September every year and followed a sinusoidal curve, decreasing to the lowest levels between December and March and showing an upward trend in April. In female individuals, the lowest concentrations were observed in December of each year, decreasing too much lower levels in those over 85 years of age (Figure 3).

Prior studies have shown that vitamin D deficiency is especially common in older women. Qun Cheng et al. showed that higher vitamin D levels are associated with male gender, rural residence, higher levels of physical activity, and education level [29]. Furthermore, Slinin et al. [30] associated lower 25-OHD levels with a higher likelihood of cognitive impairment and decline among older women. In a study of 688 patients in Turkey, Senyigit et al. [31] found that older women had lower 25-OHD levels. In future studies, it would be especially useful to evaluate these data in relation to chronic diseases, polypharmacy, socioeconomic levels, and dietary differences.

Serum 25-OHD indicates the status of vitamin D in the body, and the issue of optimal 25-OHD levels is controversial for many reasons, including controversial measurement methods [27] [28]. For example, the level of 1,25-OH2 D3 the active form of vitamin D is not correlated with actual vitamin D status and has not been found to be beneficial for clinical use [4] [32] [33]. However, most experts accept 20 μg/L of 25-OHD as the cut-off value for vitamin D deficiency.

One limitation of this study was that individuals who received vitamin D replacement therapy were not excluded. If these individuals had been excluded, it is expected that the median levels of vitamin D would have been lower. Furthermore, the Turkish Ministry of Health database does not contain any information regarding measurement methods. Therefore, the measurement methods used to obtain the study data could not be compared. Finally, patients with chronic diseases (e.g., cardiovascular disease, diabetes, and cancer) could not be excluded. Future studies should investigate potential metabolic reasons for the finding of lower 25-OHD levels in men over 85 years of age.

This is the first study with broad participation to show that the serum 25-OHD levels of the Turkish population aged 65 and older tend to decrease gradually over time. According to the results, elderly women derive less benefit from sunlight than elderly men. The most important effect of vitamin D is on bone mineralization and calcium and phosphorus metabolism. Long-term vitamin D deficiency may result in bone mineralization disorder, rickets, or osteomalacia. In addition, bone loss, osteoporosis, and fractures can be seen in cases of vitamin D insufficiency. In epidemiological studies, vitamin D deficiency has also been associated with muscle weakness, decreased physical performance, and falls. Increased vitamin D screening has led to a significant increase in 25-OHD test volume, as some countries have health policies that encourage the use of dietary supplements when 25-OHD blood levels fall within the range of 20-80 μg/L. Accordingly, it would be beneficial to reduce vitamin D-related complications, increase exposure to sunlight, develop effective methods for the intake of calcium and vitamin D-rich foods, and raise public awareness of vitamin D deficiency.

## Dodatak

### Conflict of interest statement

All the authors declare that they have no conflict of interest in this work.
